# Differences in need for antihypertensive drugs among those aware and unaware of their hypertensive status: a cross sectional survey

**DOI:** 10.1186/1471-2261-5-4

**Published:** 2005-02-03

**Authors:** Nadia A Khan, Dennis Wardman, Norman RC Campbell

**Affiliations:** 1 Department of Medicine, University of British Columbia, 620-1081 Burrard Street, St. Paul's Hospital, V6Z 1Y6, Vancouver, BC, Canada; 2 Department of Health Care and Epidemiology, Health Canada and University of British Columbia, Suite 540 – 757 W. Hastings St. V6C 3E6, Vancouver, BC, Canada; 3 Department of Medicine, University of Calgary, 3330 Hospital Drive NW, Calgary, AB, T2N 4N1, Canada

## Abstract

**Background:**

Lack of antihypertensive use among hypertensive individuals is a major public health problem. It remains unclear as to how much of this lack of treatment is because of failure to diagnose hypertension or failure to initiate drug treatment for those with a diagnosis of hypertension. The primary aim of this study was to determine the proportion of those untreated individuals who would be recommended to start drug therapy for control of blood pressure among those aware or unaware of their diagnosis of hypertension.

**Methods:**

The Canadian Heart Health Surveys (1986 – 1992), a national, cross-sectional descriptive survey (n = 23 129), was used to determine the proportion of individuals who were untreated, yet satisfied the 2004 Canadian hypertension guidelines for initiating drug therapy. Patients were divided into subgroups of those aware and unaware of having a diagnosis of hypertension according to self reported awareness from the survey.

**Results:**

Of those with untreated hypertension (= 140/90 mmHg), only 37% were aware of their diagnosis. 74% of untreated individuals aware of their diagnosis of hypertension would require drug therapy, compared to 57% of those who were unaware. Of those >65 years of age, 52% of aware individuals needed drug therapy whereas only 34% of unaware elderly would need drug treatment.

**Conclusion:**

In both unaware and aware subgroups, the majority of patients with untreated hypertension would benefit from antihypertensive drug therapy according to the 2004 Canadian Hypertension recommendations. The proportion of untreated patients that still need drug therapy was higher among those who were aware compared to those who were unaware. This finding suggests that the major gap in hypertension control may be in initiating drug therapy rather than in diagnosing hypertension. Further studies are needed to confirm these results to ultimately help strategize public health efforts in controlling hypertension.

## Background

Antihypertensive drug therapy can reduce cardiovascular morbidity and mortality by 25–30% [1-3] in those with hypertension. The 2004 Canadian hypertension guidelines [4] recommend pharmacotherapy in those patients where there is proven cardiovascular benefit from randomized controlled trial evidence. However, many of these 'at risk' patients with hypertension remain untreated even though they have a higher risk for cardiovascular events [5]. This lack of treatment is a major public health problem as hypertension is a modifiable risk factor. It remains unclear as to how much of this lack of treatment is because of failure to diagnose hypertension or failure to initiate drug treatment for those with a diagnosis of hypertension. Further understanding would help to strategize public health care initiatives in controlling high blood pressure. The primary aim of this study was to determine the proportion of those untreated individuals who would be recommended to start drug therapy for control of blood pressure among those aware or unaware of their diagnosis of hypertension.

## Methods

From the 2004 Canadian Hypertension Guidelines, pharmacotherapy is recommended if the diastolic blood pressure is > 100 mmHg; the systolic pressure is > 160 mmHg; or the diastolic blood pressure is >90 mmHg in the presence of cardiovascular heart disease or risk factors. To determine the proportions of individuals who satisfied the guidelines for initiation of drug treatment for hypertension, we used the Canadian Heart Health Surveys (1986 – 1992). This national, cross-sectional descriptive survey (n = 23 129) contains data on individual blood pressure measurements using standardized technique as well as information on cardiovascular risk profile, antihypertensive medication use and self reported awareness of hypertension. Further details of this survey have been published elsewhere [6]. We then calculated for both aware and unaware subgroups, the number of untreated hypertensive individuals that would be recommended drug therapy/ those untreated, hypertensive individuals. Patients with diabetes mellitus were excluded from the analysis because they require antihypertensive drug therapy at a much lower threshold. Descriptive statistics were used to calculate proportions and 95% confidence intervals in this study.

## Results

Of those with untreated hypertension (= 140/90 mmHg), only 37% (n = 897 786) were aware of their diagnosis. Among those aware and unaware of their diagnosis of hypertension, 35% vs. 36% were female; 14% vs. 12% had any cardiovascular disease and 31% vs. 75% were under the age of 65 years respectively. The proportions of those who would be recommended drug therapy among those who are aware and unaware are presented in Table 1. Table 1 demonstrates that compared to the unaware subgroup, a greater proportion of those aware of their hypertensive status needed drug therapy but were untreated. Among both aware and unaware groups, the largest proportion needing drug therapy was less than age 65 years. Of note, among those who were unaware, there was a considerably lower proportion that needed drug therapy over the age of 65 years.

**Table 1:** Proportions of those aware and unaware who require drug therapy (95% CI)

Awareness of hypertensive status	>65 years	18–64 years	Total
Aware	71 477/ 138 342 51.7% (51.5–51.9%)	592 186/ 759 444 78% (77.9–78.1%)	663 663/ 897 786 73.9% (73.8–74%)
Unaware	129 398/ 376 861 34% (33.8–34.2%)	738 542/ 1 143 872 64.6% (64.5–64.7%)	867 940/ 1 520 733 57.1% (57–57.2%)

## Discussion

In both unaware and aware subgroups, the majority of patients with untreated hypertension would benefit from antihypertensive drug therapy according to the 2004 Canadian Hypertension recommendations. This analysis extends the findings from other studies [5,7,8] by determining those 'at risk' individuals that would be recommended pharmacotherapy based on awareness of their hypertensive status. The proportion of untreated patients that still needed drug therapy was higher among those who were aware compared to those who were unaware. Specifically, among the elderly, most of the patients who are unaware that they are hypertensive would not actually be recommended to take drug therapy. These study findings suggest that a significant gap in hypertension control may be initiating drug therapy among those known to have hypertension. Possible barriers to initiating drug therapy may arise from patients, physicians [9] or the health care system.

There are several limitations with this study. First, the Canadian Heart Health Survey data are 12 years old and there is some evidence to suggest that prescription rates of antihypertensive drugs have improved since then [10]. However, this data set represents the best population data available on patients with hypertension in Canada since it is of high quality and captures a large cohort of Canadians. Another limitation is that the awareness or unawareness of hypertensive status is based on self report and not from physician records.

## Conclusion

This study found a considerable number of both unaware and aware patients with untreated hypertension require drug therapy. However, the majority of those who needed drug therapy were actually aware that they had a diagnosis of hypertension compared to those who were not aware. This finding suggests that the major gap in hypertension control may be in initiating drug therapy rather than in diagnosing hypertension. Further studies are needed to confirm these results to ultimately help strategize public health efforts in controlling hypertension.

## Competing interests

The author(s) declare that they have no competing interests.

## Authors' Contribution

NK, DW and NC contributed to the design of the project. NK contributed to data analysis. NK, DW and NC contributed to writing the manuscript and for substantive, intellectual editing contributions.

## Pre-publication history

The pre-publication history for this paper can be accessed here:


